# Supplementary material for: A general framework for nitrogen deposition effects on soil respiration in global forests
Source: Nat Commun. 2025 Dec 16;17:506. doi: 10.1038/s41467-025-67203-8 (PMC12804782; doi:10.1038/s41467-025-67203-8)
Supplement: Supplementary file 1 — Supplementary Information [file 41467_2025_67203_MOESM1_ESM.pdf]

Supplementary Information for

A general framework for nitrogen deposition effects on soil respiration in global forests

Xiaoyu Cen, Peter Vitousek, Nianpeng He*, Ben Bond-Lamberty, Shuli Niu, Enzai Du, Kailiang Yu, Mianhai Zheng, Kevin Van Sundert, Elizabeth L. Paulus, Liyin He, Li Xu, Mingxu Li, Klaus Butterbach-Bahl

*Corresponding author: Nianpeng He; Email: henp@igsnrr.ac.cn

This file includes:

Supplementary Texts S1 to S3

Tables S1 to S6

Figs. S1 to S10

Supplementary texts

Text S1. Response factors for quantifying the effect size of nitrogen input

There are several metrics for evaluating the effect size of nitrogen inputs (or other manipulated variables in ecological experiments such as temperature, precipitation, atmospheric CO_2 levels, etc.). Some metrics evaluate the target variable measured in the experimental plot in relative to the background value measured in the control plot, such as the “response ratio” widely used in meta-analysis [1]. Some metrics incorporate both the change in the target variable and the change in N input to account for their inherent connection, such as the “emission factor” of N-induced soil N_2O emissions [2]. With reference to how the metrics were quantified, and also based on biochemical and ecological theories, several “response factors” were defined and calculated in this study to measure the positive, negative, and integrated effects of nitrogen (N) inputs on soil respiration.

Short-term, low-level N additions often favor the growth of organisms and the decomposition of organic matter [3]. Due to the intrinsic, positive relationship between biomass and respiration (“power law”)[4], and between enzyme concentration and decomposition (“Michaelis-Menten kinetics”)[5], it is inferred that there is an inherent relationship between the amount of N added and the increased respiration (Fig S1). Therefore, we defined a “positive response factor” (f_{pos}; calculated as the increased soil respiration per unit of artificial N addition) to reveal the dose-response relationship between N input and the increased soil respiration.

High N availability exceeding a certain threshold can abruptly change community function and structure (e.g., from a N-sensitive oligotroph-dominated community to a N-tolerant copiotroph-dominated community), reducing soil respiration during the transition period [6-8]. The effect size of such an abrupt transition is often disproportionate to the N addition level triggering the change. Therefore, we defined a “negative response factor” (f_{neg}) to quantify the magnitude of the reduction, calculated as the decreased soil respiration relative to the initial soil respiration (Fig. S1).

Additionally, we defined an “integrated response factor” (f_{int}) to qualitatively measure the integrated effects of N input on soil respiration (without distinguishing the positive or negative responses; Text S2). f_{int} was calculated as the specific change in soil respiration under different levels of artificial N addition.

Text S2. Using integrated response factor to test the bimodal response of soil respiration to N input in N-limited forests

In N-limited forests, increasing N input is expected to increase, then decrease, and then increase soil respiration (Fig. 8 in the main text) because of a transition from an oligotroph-dominated to a copiotroph-dominated community. The biphasic responses of the old and new communities and the collapse of soil respiration (*SR*) during the transition period may result in a bimodal response of *SR* to N input in a N-limited forest. To test the hypothesized bimodal response, we calculated an integrated response factor using *SR* observations from experimental N addition forests that were initially N-limited ($CO_2\_exp\_NL$ dataset in the main text). Increased or decreased *SR* were not separated in this analysis because the bimodal change in *SR* was hypothesized to result from the combined positive and negative effects of N input on *SR* (see Fig. 8 in the main text for graphical illustration).

The "integrated response factor" (f_{int}; kgCO$_2$ kgN^{-1}) was calculated to reveal the change in *SR* (positive or negative) within a given range of N input. f_{int} was calculated as

$$f_{int} = \frac{(SR_2 - SR_1)}{(N_2 - N_1)} \quad \text{(Eq. S1)}$$

where SR_1 and SR_2 (unit: kgCO$_2$ ha^{-1} yr^{-1}) were the soil respiration rates observed under two different N input rates of N_1 and N_2 (kgN ha^{-1} yr^{-1}), respectively. To make the f_{int} comparable across forest sites, f_{int} was calculated within four defined N input ranges, ≤ 50 kgN ha^{-1} yr^{-1}, 50–100 kgN ha^{-1} yr^{-1}, 100–150 kgN ha^{-1} yr^{-1}, and ≥ 150 kgN ha^{-1} yr^{-1}.

With increasing N input, the integrated response factor (f_{int}) of *SR* to N input changed from positive to negative and then to positive in N-limited forests across all biomes (Fig. S6). This result suggests that *SR* in N-limited forests first increased, then decreased, and then increased in response to increasing N input in N-limited forests. The integrated, bimodal pattern of how *SR* responds to N inputs in N-limited forests aligns with the patterns revealed in separate analyses of positive and negative responses. This finding further supports the hypothesized *SR* response curve (Fig. 8 in the main text).

Text S3. Measures to address the non-independence of experimental data points

In our analysis, to avoid the over-representation of data from the same experiment (i.e., data from the same plot-year may have been analyzed and reported in different papers), we summarized data from the same year and N addition level by taking their averages. Each unique site-year-N addition level combination was used as a data point when we calculated the response factors of soil respiration (*SR*) to N addition.

Nonetheless, non-independence of the calculated response factors (as effect size metrics) could result from: (1) The shared control was used to calculate the positive response factors of *SR* to different N additions applied at the same site, so that the derived positive response factors could be correlated (*Cause 1*). (2) The effect of N addition was estimated for each experimental year so that, for a given site, the response factors corresponding to different years could be temporally correlated (*Cause 2*).

In this study, we used response factors as metrics of effect size rather than the commonly used response ratio. The response factors were used to quantify a biphasic/abrupt-transition relationship between *SR* and N inputs, instead of just deriving an integrated effect size metric showing whether N inputs increase or decrease *SR*. Although the positive and negative response factors used in this study were critical for revealing the complex, phased relationships between *SR* and N inputs, they prevented us from directly testing the influence of non-independence of data points on the revealed relationships using a meta-regression method as suggested by Gleser and Olkin [9].

However, the influence of data point non-independence could generally (and somewhat indirectly) be addressed by using an alternative effect size metric or by performing the analysis on subgroups of the dataset [10]. To account for the non-independence arising from *Cause 1*, we have defined and calculated an alternative effect size metric, integrated response factor (see Text S2 for details), to verify the revealed phased relationship between *SR* and N inputs. The integrated response factor was calculated using data from any two N addition treatments within a given range of N input rates. That is, the integrated response factors do not have a “shared control” problem like the positive response factors. Because the integrated response factors showed the same pattern as the positive and negative response factors in how *SR* responded to increasing N inputs (Fig. 8 in the main text; Fig. S6), *Cause 1* of non-independence is unlikely to challenge our finding about the response of *SR* to N inputs.

Regarding non-independence due to *Cause 2*, we have tested and validated that the revealed patterns persist regardless of using subsets of the experimental data from short-term or long-term experiments (i.e., data from experiments within/later than three years; Figs. S3, S5). Because the revealed patterns are independent of the temporal range of the experimental data used for analysis, the potential temporal correlation of the data-points is unlikely to challenge our finding about the response of *SR* to N inputs.

Supplementary tables

Table S1. Parameters of the piecewise linear regression models for the positive response factor of increased soil respiration to N input (f_{pos}). This table is supplemental to Fig. 3 in the main text and Fig. S3.

**Forest type**	**N input rate range*** (unit: kgN ha^{-1} yr^{-1})	**Model** (f_{pos} ~ N input rate)	**Parameters**
All forests (soil respiration)	x ≤ 110	y = -3.7 * x + 371.8	n = 138, R^2 = 0.23, *p* = 2e-9
	x > 110	y = -0.07 * x + 60.7	n = 113, R^2 = 0.01, *p* = 0.34
All forests (root respiration)	x ≤ 120	y = -1.1 * x + 151.7	n = 45, R^2 = 0.03, *p* = 0.22
	x > 120	y = -0.07 * x + 41.1	n = 22, R^2 = 0.01, *p* = 0.61
All forests (microbial respiration)	x ≤ 110	y = -3.2 * x + 310.4	n = 45, R^2 = 0.17, *p* = 0.005
	x > 110	y = 0.04 * x + 2.99	n = 22, R^2 = 0.02, *p* = 0.58
Tropical forests	x ≤ 120	y = -3.0 * x + 316.0	n = 40, R^2 = 0.35, *p* = 6e-5
	x > 120	y = -0.13 * x + 59.5	n = 31, R^2 = 0.10, *p* = 0.09
Temperate forests	x ≤ 110	y = -3.6 * x + 362.7	n = 71, R^2 = 0.14, *p* = 0.001
	x > 110	y = -0.08 * x + 72.4	n = 80, R^2 = 0.01, *p* = 0.42
Boreal forests	x ≤ 70	y = -10.9 * x + 615.8	n = 17, R^2 = 0.43, *p* = 0.004
	x > 70	y = -0.17 * x + 49.3	n = 12, R^2 = 0.07, *p* = 0.42
N-limited forests	x ≤ 110	y = -1.8 * x + 244.8	n = 32, R^2 = 0.11, *p* = 0.06
	x > 110	y = -0.11 * x + 51.4	n = 14, R^2 = 0.14, *p* = 0.18
N-saturated forests	x ≤ 110	y = -3.9 * x + 377.4	n = 72, R^2 = 0.26, *p* = 5e-6
	x > 110	y = -0.04 * x + 60.0	n = 74, R^2 = 0.002, *p* = 0.70
Long-term experimental forests	x ≤ 110	y = -5.8 * x + 531.6	n = 34, R^2 = 0.34, *p* = 3e-4
	x > 110	y = -0.16 * x + 71.4	n = 25, R^2 = 0.05, *p* = 0.29

* Change points of N input rates were determined with reference to Fig. S2.

Table S2. Parameters of the constructed random forest model using the *CO_2_exp* dataset. This model was used to derive the partial dependence of negative response factor (f_{neg}) on N input, and to assess the relative importance of environmental factors on f_{neg} (Fig. 4 in the main text).

Model	f_{neg} ~ MAT + MAT.cv + MAP + MAP.cv + N_{dep} + N_{dep}.cv + Sand + Clay + pH + SOC + TN + N_{in}	
Parameters	Mtry	4
	Ntree	500
	Number of runs	1000
	R^2	0.34

f_{neg}: negative response factor of decreased soil respiration to N input; MAT: mean annual temperature; MAP: mean annual precipitation; N_{dep}: mean annual N deposition; Sand: soil sand content; Clay: soil clay content; MAT.cv, MAP.cv and N_{dep}.cv are the corresponding coefficients of temporal variation; pH: soil pH; SOC: soil organic carbon content; TN: soil total nitrogen content; N_{in}: N input rates in the experimental plots (including artificial N addition). The predictors were selected based on mechanistic relevance and data availability.

Table S3. Frequencies of soil respiration being decreased by different N addition levels.

	N addition rate (kgN ha^{-1} yr^{-1})					
	≤15	15–50	50–100	100–150	150–200	>200
No. site-year-N addition level combinations where soil respiration was decreased by N input (n(ΔSR_N <0))	0	38	40	36	5	21
No. site-year-N addition level combinations where soil respiration was increased by N input (n(ΔSR_N ≥0))	7	78	48	31	14	14
Frequency of soil respiration being decreased (= n(ΔSR_N <0) / n(ΔSR_N))	0	0.33	0.45	0.54	0.26	0.60

Table S4. Parameters of the constructed random forest regression model using *CO_2_obs* dataset. The model was used to predict the soil respiration rates in global forests and to derive the partial dependence of soil respiration on N deposition.

Model	SR ~ MAT + MAT.cv + MAP + MAP.cv + N_{dep} + N_{dep}.cv + Sand + Clay + pH + SOC + TN + s_N	
Parameters	Mtry	4
	Ntree	500
	Number of runs	1000
	R^2	0.51
	Correlation coefficient between observed and predicted SR	0.71

SR: soil respiration rate under natural conditions; MAT: mean annual temperature; MAP: mean annual precipitation; N_{dep}: mean annual N deposition; Sand: soil sand content; Clay: soil Clay content; MAT.cv, MAP.cv and N_{dep}.cv are the corresponding coefficients of temporal variation; pH: soil pH; SOC: soil organic carbon content; TN: soil total nitrogen content; s_N: sensitivity of soil N_2O emission to N deposition, which indicates soil N limitation or saturation status. The predictors were selected based on mechanistic relevance and data availability.

Table S5. Response factors and key parameters for estimating the contribution of N deposition to global forest soil respiration budget. This table is supplemental to Table 1 in the main text.

Biome	N status	Area (10^8ha)	f_{pos} ($kgCO_2$ kgN^{-1})	f_{neg} ($kgCO_2$ $kgCO_2^{-1}$)	P_{dec} (%)	$Area_{dec}$ (10^8ha)
Tropical	N-limited	8.4	238.0 (57.3)	-0.09 (0.03)*	1.5	0.12
	N-saturated	9.6	349.4 (55.9)	-0.09 (0.03)	4.9	0.47
	Subtotal	18.0	299.0 (56.6)	-0.09 (0.03)	3.3	0.59
Temperate	N-limited	3.6	235.3 (57.4)	-0.08 (0.02)	3.3	0.12
	N-saturated	3.8	336.8 (56.3)	-0.14 (0.02)	6.9	0.26
	Subtotal	7.4	278.0 (56.9)	-0.1 (0.02)	5.1	0.38
Boreal	N-limited	10.0	241.2 (57.3)	-0.03 (0.01)*	0.7	0.07
	N-saturated	3.0	367.6 (55.7)	-0.03 (0.01)	2.0	0.06
	Subtotal	13.0	266.6 (56.9)	-0.03 (0.01)	1.0	0.13
Total		38.4	280.3 (56.8)	-0.07 (0.02)	2.9	1.10

f_{pos}: positive response factor of increased soil respiration to the current level of N deposition; f_{neg}: negative response factor of decreased soil respiration to the current level of N deposition; P_{dec}: probability of soil respiration being decreased by the current level of N deposition; $Area_{dec}$: area of forests where soil respirations were decreased by the current level of N deposition.

* No data corresponding to this biome-N status combination, so all data from this biome were used to derive the parameter instead.

Table S6. Parameters for selecting the optimal non-linear regression model for P_{dec}, the probability of soil respiration being decreased by N inputs.

Model	Formula*	Log likelihood	AIC
Weibull model (type 1)	$f(x) = \exp(-\exp(b(\log(x) - e)))$	33.44564	-52.89129
Log-logistic model	$f(x) = \frac{1}{1 + \exp(b(\log(x) - \log(e)))}$	32.48269	-50.96538
Weibull model (type 2)	$f(x) = c + (d - c)(1 - \exp(-\exp(b(\log(x) - \log(e)))))$	31.44989	-48.89979
Michaelis-Menten model	$f(x, (c, d, e)) = c + \frac{d - c}{1 + (e/x)}$	40.23633	-60.47266

* Formula were from Ritz, et al. [11].

Supplementary figures

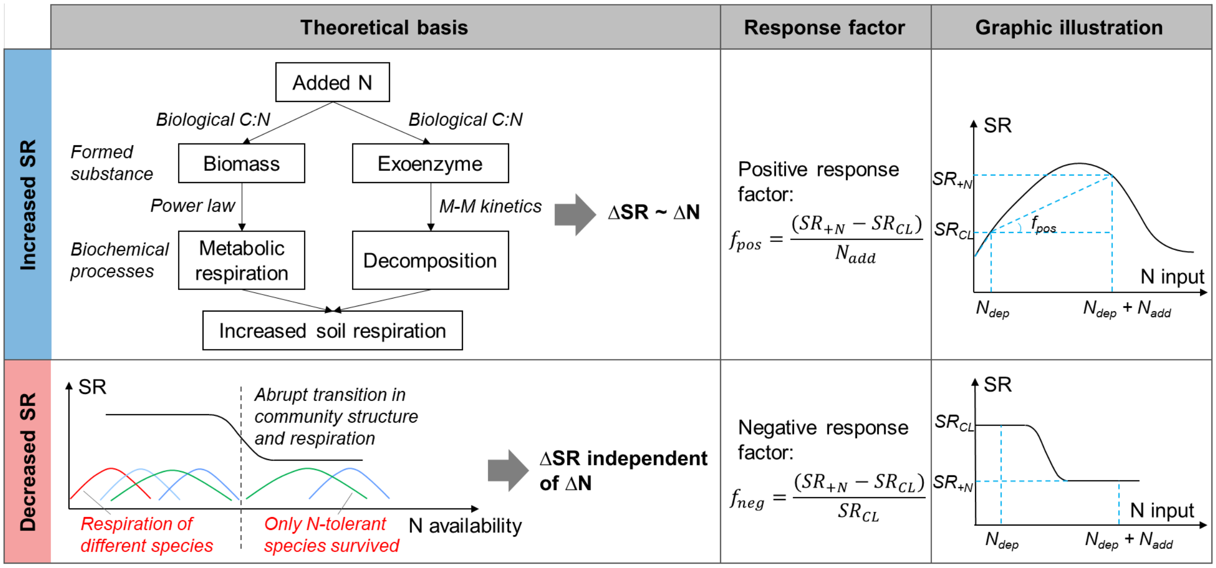

Fig. S1 Theoretical basis and graphical illustration of the positive response factor (f_{pos}) and negative response factor (f_{neg}) of soil respiration to N inputs. N_{dep} is the atmospheric N deposition rate. SR_{CL} is the soil respiration rate in the control plot receiving background N deposition only. SR_{+N} is the soil respiration rate observed in the experimental plot receiving artificial N addition. N_{add} is the N addition rate.

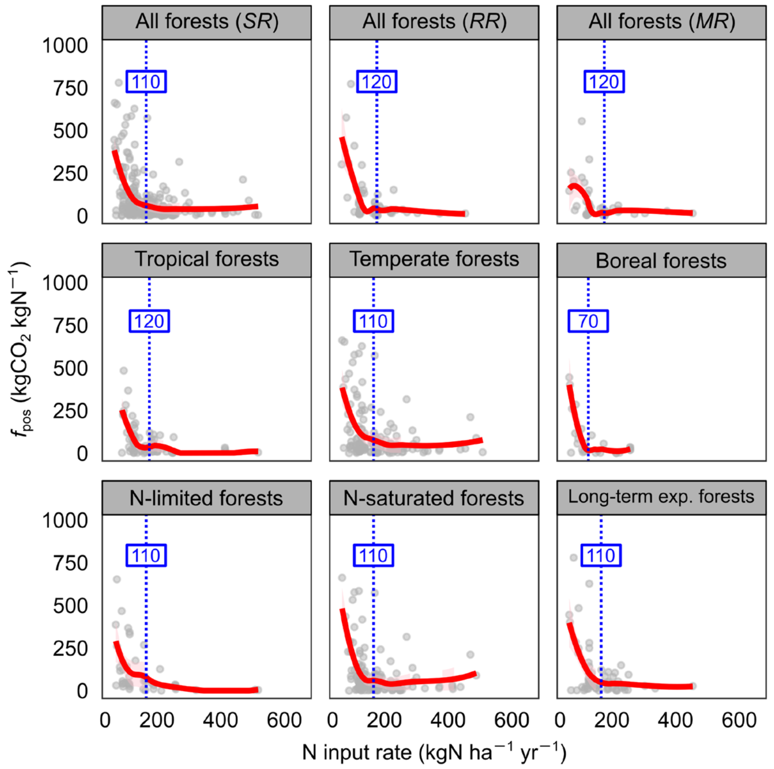

Fig. S2 Local polynomial regression (LOESS) model for the response of increased soil respiration to N input (f_{pos}) in forests. Red lines indicate the LOESS models, and pink shadings indicate the standard errors of the models. Blue dashed lines indicate the thresholds used for piecewise regression models (Table S1, Fig. 3, Fig. S3), which were determined based on the change points in the LOESS curves and the goodness of fit of the derived piecewise regression models.

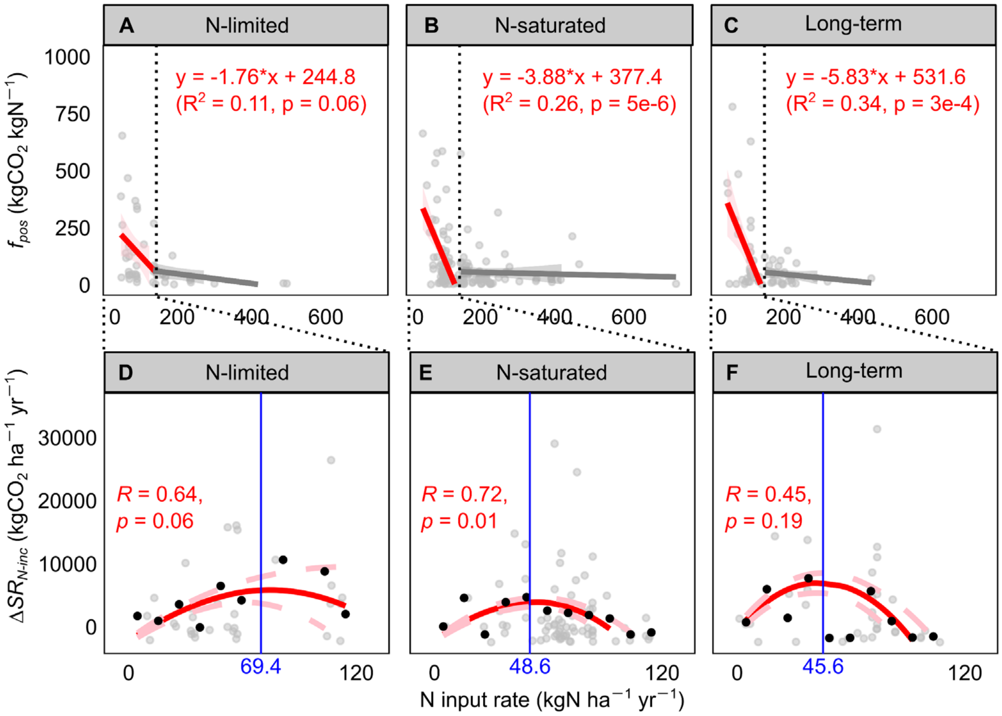

Fig. S3 Increased soil respiration ($\Delta SR_{N\text{-}inc}$) by N inputs in forests (supplemental to Fig. 3 in the main text). (**A–C**) Piecewise linear regression models relating N input rate to the response factor of increased *SR* by N input (f_{pos}) in experimental forests. Gray points show the observations of f_{pos} derived from experimental dataset (*CO2_exp*). Red (gray) lines and font show the fitted linear models for f_{pos} and low (high) N input rate. The linear models for f_{pos} and low N input rate (red font) were then used to infer the quadratic models for $\Delta SR_{N\text{-}inc}$. (**D–F**) Quadratic models for $\Delta SR_{N\text{-}inc}$ in forests. Gray points show the observed $\Delta SR_{N\text{-}inc}$ in experimental forests. Black points show the moving average of $\Delta SR_{N\text{-}inc}$ for every 10 kgN ha^{-1} yr^{-1}; this aggregation of data was to mitigate the influence of extreme values. The red lines show the quadratic models calculated from the linear models. Pink dashed lines show the range of uncertainties of the quadratic models. Red font show the correlation coefficient (*R*) between the observed and predicted mean $\Delta SR_{N\text{-}inc}$. Blue lines and font show the turning points of the quadratic models. “N-limited” forests refer to experimental forests which were initially N-limited and where N addition experiments had been conducted for no more than 3 years; “N-saturated” forests refer to experimental forests which were initially N-saturated and where N addition experiments had been conducted for no more than 3 years; “long-term” forests refer to those where N addition experiments had been conducted for more than 3 years, regardless of the initial N status.

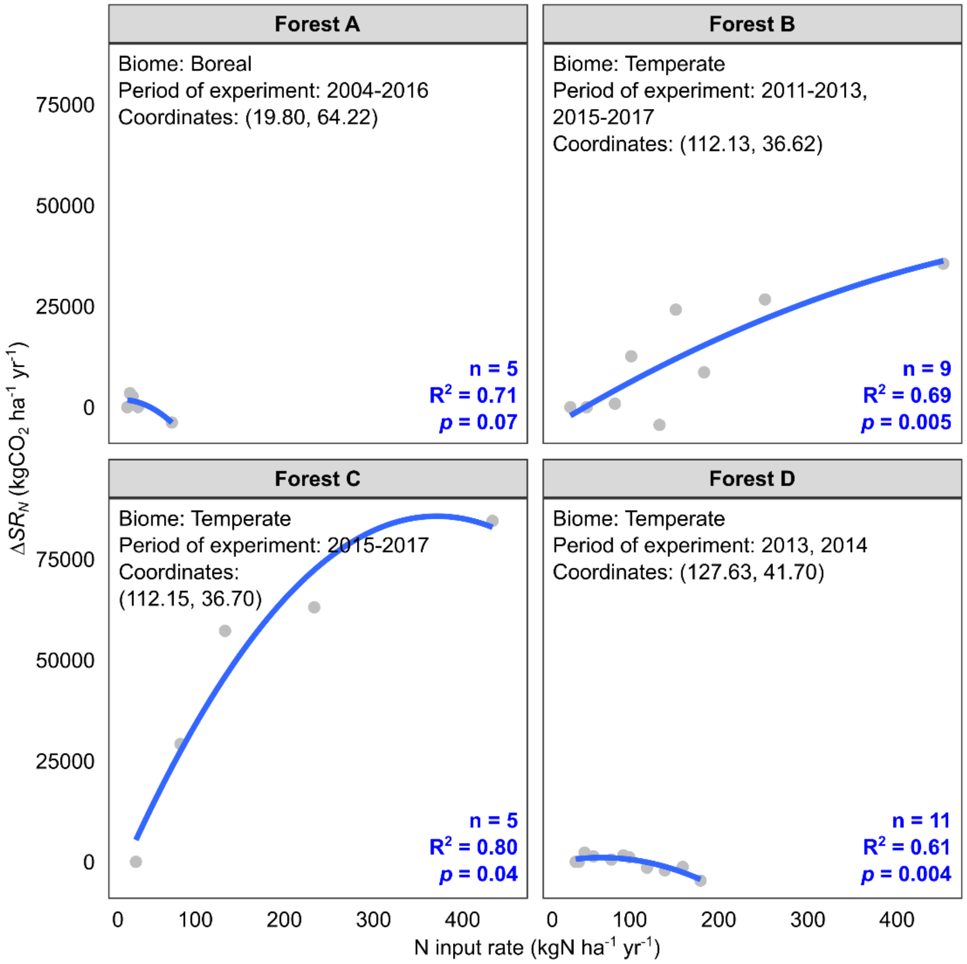

Fig. S4 Relationship between N input rate and the N-induced change in soil respiration rate (ΔSR_N) in four experimental forests with at least five N input levels (including the control). In each panel, a gray point represents one N addition level and the corresponding ΔSR_N. Here, ΔSR_N was calculated as the difference in *SR* in paired control and experimental N addition plots. The blue curves show the quadratic models fitted to the experimental data of each forest. Blue fonts show the goodness of fit of the quadratic models. Black fonts show the basic information for each experimental forest. The information was retrieved from the original sources of data: Forest A [12], Forest B [13,14], Forest C [15], Forest D [16,17].

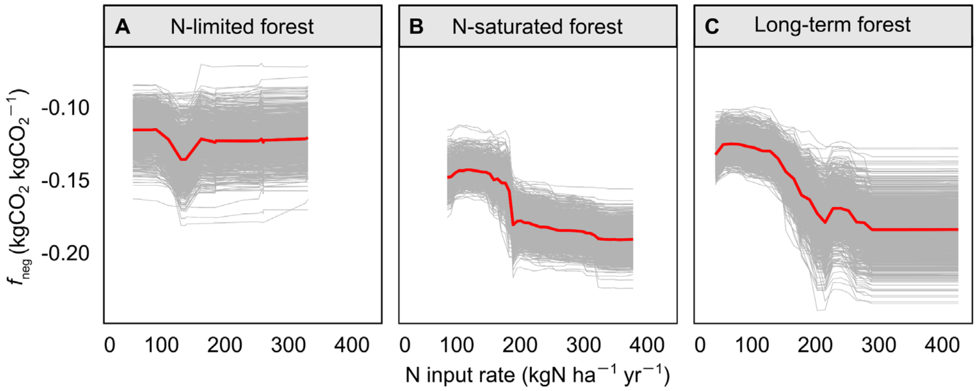

Fig. S5 Decreased soil respiration (*SR*) by N inputs in forests (supplemental to Fig. 4 in the main text). The negative response factor (f_{neg}) of decreased soil respiration to N input was used to indicate the magnitude of the decrease. In panels A to C, gray curves show partial dependence plots derived from 1000 random forest models built using 1000 different subsets of the experimental data (to avoid that the derived pattern was driven by a few observations), and red curve shows the arithmetic mean of the 1000 gray curves.

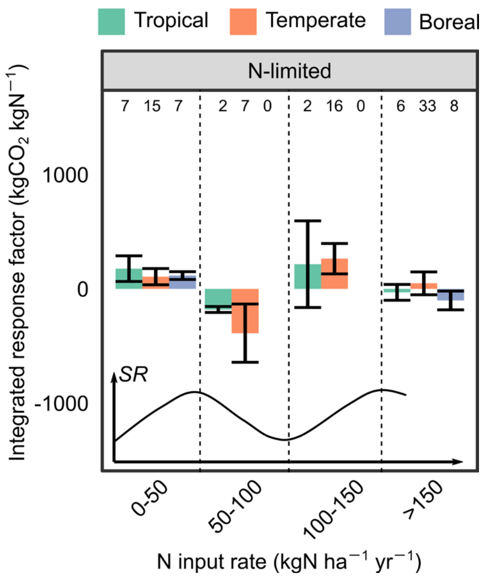

Fig. S6 Integrated response factors of soil respiration rate to N input (f_{int}) calculated for four different N input levels in N-limited experimental forests. Columns indicate the mean f_{int} corresponding to each N addition level in each biome; error bars indicate the standard errors of the mean values. Numbers on the top of the panel are the total number of calculated f_{int} values corresponding to each N addition level in each biome.

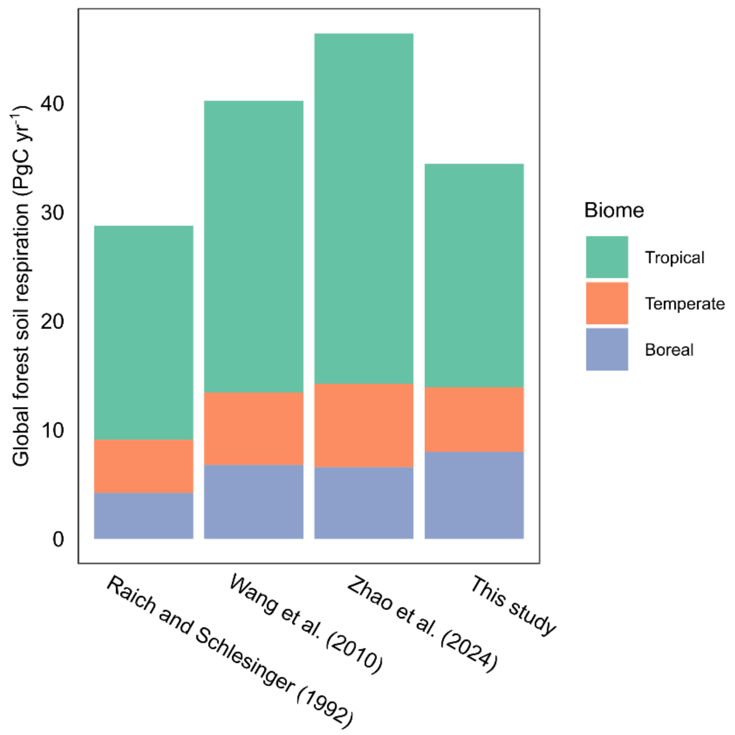

Fig. S7 Comparing the estimated global forest soil respiration budgets in this study with those from previous studies [18-20]. Different colors refer to the forest soil respiration budgets of different biomes.

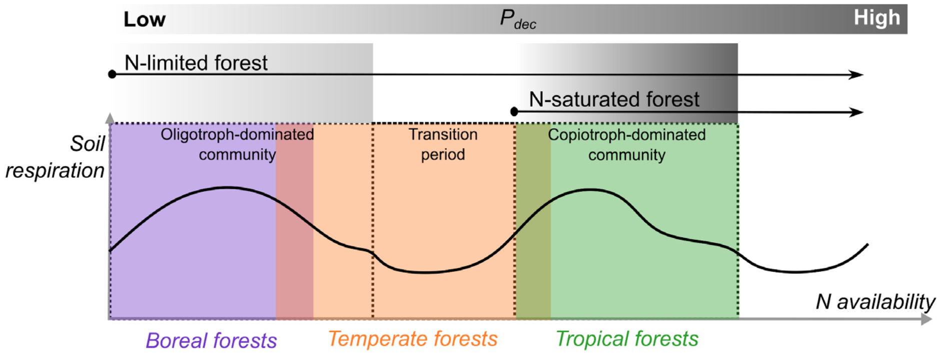

Fig. S8 Hypothesized soil respiration responses to increasing nitrogen availability in forests in different biomes. Background nitrogen status differences contribute to the variation in forest soil respiration responses across biomes (Figs. 3–6 and Table 1 in the main text).

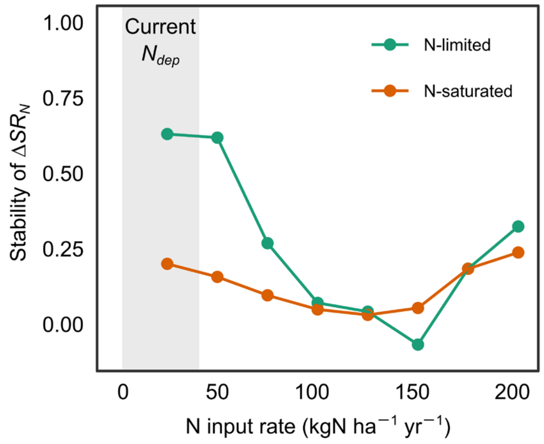

Fig. S9 Stability index of N-induced soil respiration change (Δ*SR*$_N$) in N-limited and N-saturated forests. The stability index of Δ*SR*$_N$ was calculated as the inverse of CV (coefficient of variation) of the observed soil respiration changes corresponding to a N addition level. Gray shading indicates the range of the current N deposition rates in the majority of forests, which is 0–40 kgN ha^{-1} yr^{-1}. As long as the N deposition rate does not exceed 100 kgN ha^{-1} yr^{-1}, the stabilities of Δ*SR*$_N$ in both N-limited and N-saturated forests tend to decrease under increasing N deposition rate.

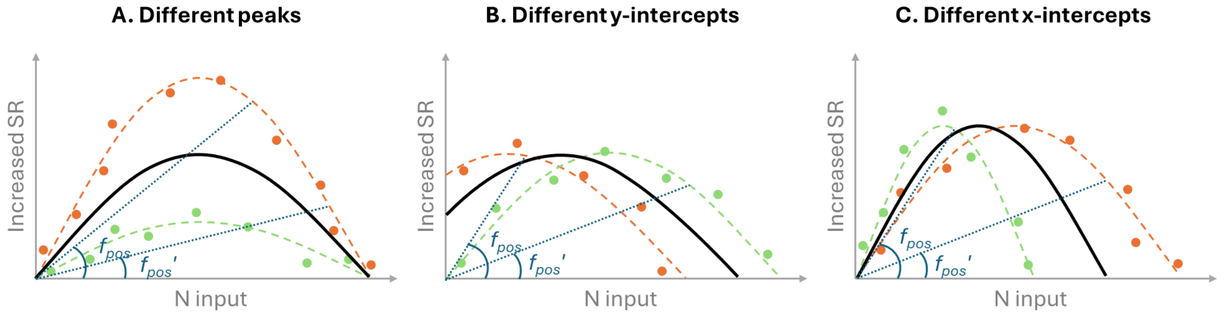

Fig. S10 Potential causes that may weaken the quadratic relationship between N inputs and increased soil respiration in a data synthesis analysis. In panels A–C, points of the same color show experimental data from the same site, and dashed curves of the corresponding color show the site-specific quadratic curve. The black solid curve shows the fitted quadratic curve using data from all sites. As can be seen, the goodness of fit of the black curves is not as good as that of the site-specific colored curves, due to the different peaks and intercepts of the site-specific curves. However, with increasing N input, the positive response factor of increased soil respiration to N input (f_{pos}; see Text S1 for detailed explanation and illustration) decreases regardless of whether the model was fitted for each site or for all sites combined (see the change from f_{pos} to f_{pos}' in the panels). Therefore, a significant negative relationship between f_{pos} and N input was found in all forests (Fig. 3a–c, g–i in the main text), despite the differences between sites.

References

1 Hedges, L. V., Gurevitch, J. & Curtis, P. S. The meta-analysis of response ratios in experimental ecology. *Ecology* **80**, 1150-1156, doi:10.1890/0012-9658(1999)080[1150:TMAORR]2.0.CO;2 (1999).

2 Leip, A., Busto, M. & Winiwarter, W. Developing spatially stratified N2O emission factors for Europe. *Environ Pollut* **159**, 3223-3232, doi:10.1016/j.envpol.2010.11.024 (2011).

3 Fog, K. THE EFFECT OF ADDED NITROGEN ON THE RATE OF DECOMPOSITION OF ORGANIC MATTER. *Biol Rev* **63**, 433-462, doi:10.1111/j.1469-185X.1988.tb00725.x (1988).

4 Reich, P. B., Tjoelker, M. G., Machado, J.-L. & Oleksyn, J. Universal scaling of respiratory metabolism, size and nitrogen in plants. *Nature* **439**, 457-461, doi:10.1038/nature04282 (2006).

5 Michaelis, L. & Menten, M. L. Die kinetik der invertinwirkung. *Biochem. z* **49**, 352 (1913).

6 Egidi, E., Coleine, C., Delgado-Baquerizo, M. & Singh, B. K. Assessing critical thresholds in terrestrial microbiomes. *Nature Microbiology* **8**, 2230-2233, doi:10.1038/s41564-023-01536-2 (2023).

7 Zheng, M. *et al.* Temporal patterns of soil carbon emission in tropical forests under long-term nitrogen deposition. *Nat Geosci* **15**, 1002-1010, doi:10.1038/s41561-022-01080-4 (2022).

8 Bobbink, R. *et al.* Global assessment of nitrogen deposition effects on terrestrial plant diversity: a synthesis. *Ecol Appl* **20**, 30-59, doi:10.1890/08-1140.1 (2010).

9 Gleser, L. J. & Olkin, I. Stochastically dependent effect sizes. *The Handbook of Research Synthesis and Meta-analysis*, 357-376 (2009).

10 Noble, D. W. A., Lagisz, M., O'Dea, R. E. & Nakagawa, S. Nonindependence and sensitivity analyses in ecological and evolutionary meta-analyses. *Molecular Ecology* **26**, 2410-2425, doi:10.1111/mec.14031 (2017).

11 Ritz, C., Baty, F., Streibig, J. C. & Gerhard, D. Dose-response analysis using R. *Plos One* **10**, e0146021, doi:10.1371/journal.pone.0146021 (2016).

12 Forsmark, B., Nordin, A., Maaroufi, N. I., Lundmark, T. & Gundale, M. J. Low and High Nitrogen Deposition Rates in Northern Coniferous Forests Have Different Impacts on Aboveground Litter Production, Soil Respiration, and Soil Carbon Stocks. *Ecosystems* **23**, 1423-1436, doi:10.1007/s10021-020-00478-8 (2020).

13 Yu, H. *Effects of changing soil acidity/alkalinity on soil nitrogen and greenhouse gas fluxes in a Pinus tabulaeformis forest in Taiyue*, Beijing Forestry University, (2019).

14 Zhao, B. *Soil Respiration in Response to Thinning and Simulated Nitrogen Deposition in Pinus Tabuliformis Forest in Taiyue Mountain*, Beijing Forestry University, (2019).

15 Yu, H., Chen, Y., Zhang, H. & Zhou, Z. The effect of inorganic nitrogen addition on soil nitrogen and greenhouse gas flux for the Pinus tabulaeformis forest in Taiyue Mountain, Shanxi Province. *Journal of Nanjing Forestry University* **62**, 85 (2019).

16 Peng, B., Sun, J. F., Liu, J., Xia, Z. W. & Dai, W. W. Relative contributions of different substrates to soil N2O emission and their responses to N addition in a temperate forest. *Sci Total Environ* **767**, 8, doi:10.1016/j.scitotenv.2020.144126 (2021).

17 Geng, J. *et al.* Soil nitrate accumulation explains the nonlinear responses of soil CO2 and CH4 fluxes to nitrogen addition in a temperate needle-broadleaved mixed forest. *Ecol Indic* **79**, 28-36, doi:10.1016/j.ecolind.2017.03.054 (2017).

18 Raich, J. W. & Schlesinger, W. H. The global carbon dioxide flux in soil respiration and its relationship to vegetation and climate. *Tellus B* **44**, 81-99, doi:10.1034/j.1600-0889.1992.t01-1-00001.x (1992).

19 Wang, W., Chen, W. & Wang, S. Forest soil respiration and its heterotrophic and autotrophic components: Global patterns and responses to temperature and precipitation. *Soil Biology and Biochemistry* **42**, 1236-1244, doi:10.1016/j.soilbio.2010.04.013 (2010).

20 Zhao, Z., Ding, X., Wang, G. & Li, Y. 30 m Resolution Global Maps of Forest Soil Respiration and Its Changes From 2000 to 2020. *Earth's Future* **12**, e2023EF004007, doi:10.1029/2023EF004007 (2024).
